# Race, ethnicity and mortality in the United States during the first year of the COVID-19 pandemic: an assessment

**DOI:** 10.1007/s44155-022-00019-9

**Published:** 2022-09-26

**Authors:** Seife Dendir

**Affiliations:** Radford University, Radford, VA 24142 USA

**Keywords:** COVID-19, Mortality, Race, Ethnicity, Disparities, United States

## Abstract

The fact that a disproportionate share of the disease burden from COVID-19, including mortality, has been borne by racial and ethnic minority communities is well documented. The purpose of this paper is to reassess the “race/ethnicity effect” in COVID-19 mortality in the United States. Using an ecological regression framework and county-level data, the study aims to make two contributions. First, it estimates the race/ethnicity effect for *all* major racial/ethnic groups at *four* important junctures during the first year of the pandemic. In doing so, it seeks to provide the fullest possible picture of the nature and evolution of the race/ethnicity effect. Second, it estimates the race/ethnicity effect net of basic socioeconomic factors (SEF). This helps to identify the likely mechanisms through which the race/ethnicity effect operates. Racial/ethnic composition is flexibly measured in two ways—by percentage contributions to county population and by indicators of group plurality. The ecological regressions revealed a positive association between the size of three racial/ethnic groups—non-Hispanic Blacks, non-Hispanic American Indian and Alaskan Natives (AIAN) and Hispanics—and county mortality, although the association was stronger and more consistent for Blacks and AIANs. Furthermore, accounting for basic SEF had different impacts on the race/ethnicity-mortality association for the three groups. For Hispanics, it was almost fully mediated. For Blacks, it decreased but remained statistically significant [62–6% higher mortality associated with a 1-standard deviation increase in Black share of county population; 2.3–1.1 times higher mortality in the average Black plurality county]. For AIANs, it was largely unaffected or even increased [44–10% higher mortality associated with a 1-standard deviation increase in AIAN share; 6.2–1.8 times higher mortality in AIAN plurality county). For all groups, the race/ethnicity effect generally decreased as the pandemic wore on during the first year.

**Supplementary Information:**

The online version contains supplementary material available at 10.1007/s44155-022-00019-9.

## Introduction

COVID-19 has had a disproportionate impact on racial and ethnic minority communities in the United States. Early in the pandemic, The New York Times noted that “The coronavirus is infecting and killing black people …at disproportionately high rates” [1]. Six months into the pandemic, as of August 2020, the Centers for Disease Control and Prevention (CDC) documented that American Indian or Alaska Native (AIAN) and Hispanic persons were experiencing the highest infection rates, at 2.8 times the rate for non-Hispanic Whites. The infection rate of Black or African Americans was 2.6 times higher than Whites. Blacks were also dying at twice the rate of Whites, followed by AIANs (1.4 times) and Hispanics (1.1 times) [2]. As of March 2021, a year into the pandemic and more than three months after the rollout of COVID-19 vaccinations, the disease burden, particularly mortality, remained markedly high in minority communities. Nationwide, mortality (deaths per 100,000 people) was highest among Blacks (178), followed by AIANs (172), Hispanics (154), Native Hawaiian or Other Pacific Islanders (NHPIs; 144), and Whites (124) [3].

It is generally recognized that racial/ethnic differences in COVID-19 mortality were broadly manifesting long-term structural inequities, whether in education, employment, housing, healthcare or the criminal justice system [4–7]. Some of the effects of these inequities were more direct and immediate. People of color are overrepresented in frontline and public-facing occupations—in healthcare and other service industries—that increased their exposure to the virus [8, 9]. They are also more likely to live in overcrowded, multi-family dwellings, which facilitated the transmission of the disease [10], and in areas that record higher levels of long-term air pollution [11, 12]. Exposure to pollution has been shown to be a significant factor in serious complications and fatality from COVID-19 [13]. But many of the structural inequities also caused these communities to have poor health generally and to be burdened by specific comorbidities. African Americans, for example, suffer disproportionately from comorbidities such as obesity, hypertension, diabetes mellitus and heart disease, all of which are associated with more severe COVID-19 outcomes [11, 14, 15].

A majority of the early reports and studies on the pandemic’s uneven impacts reported and analyzed the disparities in cases and disease outcomes (e.g. hospitalizations, deaths) by race/ethnicity (see for example, [16–18]). While informative and generally insightful, this type of bivariate analysis does not account for well-known socioeconomic and environmental correlates, thereby failing to shed much light on the mechanisms through which the race/ethnicity effect operates. Later studies did investigate the degree to which the race/ethnicity effect in COVID-19 outcomes is mediated by different factors, from social determinants of health [19–22] to structural racism [4, 23]. But a majority of these studies typically focused on a single racial/ethnic group (e.g. African Americans) and analyzed the race/ethnicity effect at a single point in time during the pandemic.

The purpose of this study is two-fold. First, in its approach, the paper aims to provide the a more complete picture of the race/ethnicity effect in COVID-19 mortality in the U.S. To do this, it assesses the effect for *all* racial/ethnic groups (as classified by the U.S. Census Bureau) and *over* time (across four dates in the first 13 months of the pandemic). There have been calls for a more encompassing analysis of the race/ethnicity effect that considers all groups and captures the evolution of racial/ethnic disparities in COVID-19 outcomes over time [18, 19, 21, 24]. Second, in its analysis, the study estimates the race/ethnicity effect “net” of basic socioeconomic factors (henceforth SEF), namely poverty, employment, income and education. By explicitly considering the role of SEF in racial/ethnic disparities in COVID-19 mortality, the results of the analysis would be instructive for policy. If, for example, much of the race/ethnicity effect turns out to be mediated by basic SEF, addressing structural inequities in these areas through targeted policies will help avert similar outcomes in a future health crisis (not to mention the imperative of addressing each dimension of inequity in and of itself).

The data required to conduct multivariate analysis of this type are rather profound. Ideally one would use a person-level dataset that maps disease outcome to clinical background and demographic and socioeconomic information [25]. Data records of this kind are unavailable in the U.S. except in some specific clinical settings (e.g. from a particular health provider; even then the socioeconomic data are typically very thin) [26]. In fact, researchers have generally lamented the lack of systematic collection and reporting of disaggregated race/ethnicity data on COVID-19 nationally [27, 28]. Except for a handful of states that report county-level data, mortality by race/ethnicity and related demographics in the U.S. are typically reported at the state level only [3]. Given the data shortcomings, this study will use county-level data in an ecological regression framework [11, 13]. Specifically, county-level COVID-19 mortality will be regressed on measures of county racial/ethnic composition, basic SEF, and a set of covariates.

## Data

The data for the study come from various sources, all of which are publicly available. County-level cumulative deaths are obtained from The New York Times and are based on reports from state and local health agencies. The Times dataset documents cumulative cases and deaths beginning with the first reported case in the U.S. on January 21, 2020 (the first documented death was on February 29, 2020). The population and race/ethnicity data are from the Census Bureau’s Annual County Resident Population Estimates (2010–2018) and are sourced from the dataset put together by Killeen et al. [29]. The demographic and socioeconomic variables are obtained from the Social Vulnerability Index (SVI) database (2018) of the Centers for Disease Control and Prevention/Agency for Toxic Substances and Disease Registry (CDC/ATSDR). The underlying data are based on the U.S. Census Bureau’s five-year (2014–18) American Community Survey (ACS). The environmental variable, which measures county-level pollution using estimates of fine particulate matter in the air (PM_2.5_), comes from Wu et al. [13].

**Fig. 1:**
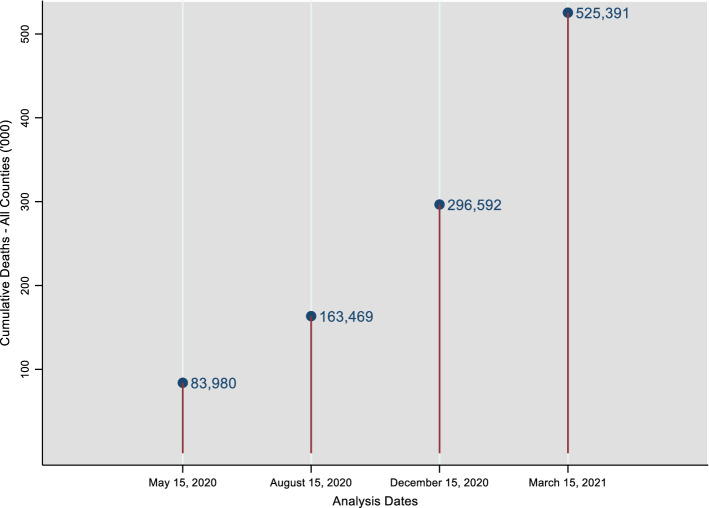
All-county cumulative deaths across analysis timeline

Because one of the aims of this study is to document and analyze how the relationship between race/ethnicity and COVID-19 mortality evolved during the course of the pandemic, four dates during roughly the first year of the pandemic are chosen for analysis. Each of these dates is associated with some kind of a milestone. The dates are: May 15, 2020 (toward the end of the first wave), August 15, 2020 (toward the end of the second wave), December 15, 2020 (a day after the rollout of the vaccination program), and March 15, 2021 (almost exactly a year after the World Health Organization declared COVID-19 a global pandemic and the U.S. declared a national emergency).^1^ All U.S. counties for which cumulative deaths are reported for at least one of the above dates and for which data on the other variables are not missing are included in the analysis. Puerto Rico does not report COVID-19 deaths at the county (municipio) level and is not part of this study. Alaska is also excluded because it lacks the air quality data. Because The New York Times dataset combined the five boroughs of New York City into a single area for reporting COVID-19 related statistics, data from the New York City Department of Health and Mental Hygiene at the borough/county level are used instead. Figure 1 presents cumulative deaths in all sample counties across the analysis timeline.

Table 1 presents summary statistics on the relevant county-level variables that are used in the regression exercises. As of May 15, 2020, county COVID-19 mortality, measured as deaths per 100,000 people, averaged 10.7, which rose to 28.9 as of August 15, approximately six months into the pandemic. At the beginning of the vaccination campaign, average county deaths reached almost 100 (98.6). As of March 15, 2021, a year from the official declaration of a national emergency in the U.S., county-level mortality averaged 180.5. The distribution of deaths was highly skewed at the beginning of the pandemic, with mean mortality at five times the median. As the pandemic spread across the country, the skewness declined considerably—a year into the pandemic, the mean was only about 1.1 times the median. Even so, there was significant variation in county mortality and about 2 percent of counties recorded zero deaths.

**Table 1:** Summary statistics on important variables

Variable	N	Mean	Std. Dev	Min	Max	25th Perc	Median	75th Perc
Deaths (per 100,000)								
May 15, 2020	2900	10.69	24.21	0	307.3	0	2.046	9.738
August 15, 2020	3116	28.87	42.24	0	420.4	2.000	13.58	38.07
December 15, 2020	3136	98.59	79.93	0	765.7	43.35	81.12	131.8
March 15, 2021	3136	180.5	110.0	0	842.3	103.9	165.8	235.0
Age ≥ 65 (%)	3136	18.37	4.582	3.800	55.60	15.50	18	20.80
Age ≤ 17 (%)	3.136	22.36	3.492	5.300	40.50	20.30	22.30	24.10
Poverty (%)	3136	15.61	6.475	2.300	55.10	11	14.70	19.10
Unemployment rate (%)	3136	5.767	2.845	0	28.90	4	5.400	7.100
Per Capita Income	3136	27,029	6,506	10,148	72,832	22,764	26,244	30,103
No High School Diploma (%)	3136	13.41	6.340	1.200	66.30	8.800	12.10	17.20
Uninsured rate (%)	3 136	10.06	5.077	1.700	45.60	6.200	9.200	12.60
Pollution (PM_2.5_)	3091	8.406	2.520	2.060	15.79	6.341	8.793	10.49
Race/ethnicity (% share)								
White	3136	76.08	20.11	2.691	97.89	64.40	83.39	92.31
Black	3136	9.008	14.31	0	85.41	0.708	2.240	10.28
AIAN	3136	1.891	7.267	0	90.51	0.243	0.385	0.776
Asian	3136	1.493	2.886	0	42.79	0.425	0.674	1.339
NHPI	3136	0.105	0.955	0	48.86	0.0182	0.0362	0.0684
Mixed	3136	1.804	1.304	0	23.77	1.162	1.521	2.023
Hispanic	3136	9.620	13.77	0.610	96.36	2.399	4.390	9.998
Race/ethnicity (Largest group dummy)								
White	3136	0.908	0.289	0	1	1	1	1
Black	3136	0.0408	0.198	0	1	0	0	0
AIAN	3136	0.0105	0.102	0	1	0	0	0
Asian/NHPI	3136	0.00223	0.0472	0	1	0	0	0
Hispanic	3136	0.0386	0.193	0	1	0	0	0

The racial/ethnic variables are: non-Hispanic White alone (White), Black or African American alone (Black), American Indian and Alaska Native alone (AIAN), Asian alone (Asian), Native Hawaiian and Other Pacific Islander alone (NHPI), Two or more races (mixed); and Hispanic

For the purposes of this study, county racial/ethnic composition is measured in two ways. The first is by the percentage contribution of each racial/ethnic group to county population. Following the standard classification adopted by the U.S. Census Bureau, all racial/ethnic groups are considered. These are: non-Hispanic White alone (White); non-Hispanic Black or African American alone (Black); non-Hispanic American Indian and Alaska Native alone (AIAN); non-Hispanic Asian alone (Asian); non-Hispanic Native Hawaiian and Other Pacific Islander alone (NHPI); non-Hispanic Two or More Races (mixed); and Hispanic. Looking at the racial/ethnic compositions in Table 1, in the average county, Whites comprised three-quarters of the population, followed by Hispanics and Blacks, at 9.6 and 9 percent, respectively. AIANs contributed to about 1.9 percent of county population, on average, about the same as that of people with two or more races (1.8 percent).

The second measure stratifies counties according to their largest racial/ethnic group (that is, plurality group) and constructs dummy (or indicator) variables. A dummy variable equals 1 if the largest share of a county’s population comes from a given racial/ethnic group, and is zero otherwise. For example, the dummy *Largest_Black* is equal to 1 for counties where Blacks comprise the largest racial group in a county, zero otherwise. *Largest_AIAN* and *Largest_Hispanic* are similarly defined for counties where AIANs and Hispanics comprise the largest racial category, respectively. Due to the small number of counties where Asians or NHPIs individually comprise the largest racial group, a single dummy variable is defined for counties where either group is the largest (*Largest_ANHPI*). No county in the U.S. has Two or More Races (mixed) as the largest racial group. The summary statistics in Table 1 show that Whites comprise the largest group in 91 percent of U.S. counties. Blacks and Hispanics each made up the largest group in roughly the same proportion of counties (4.1 and 3.9 percent, respectively). Only 1.1 percent of counties had AIANs as the largest racial/ethnic group.

**Fig. 2:**
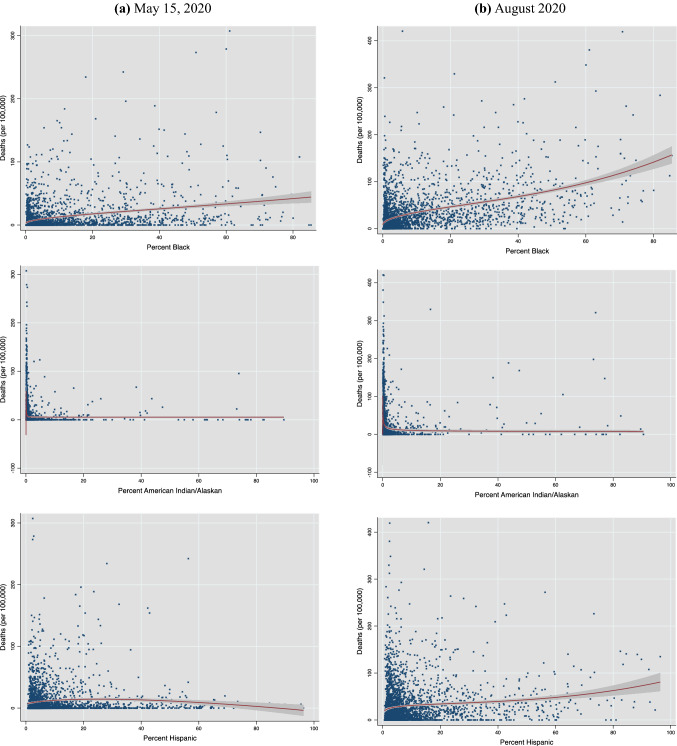

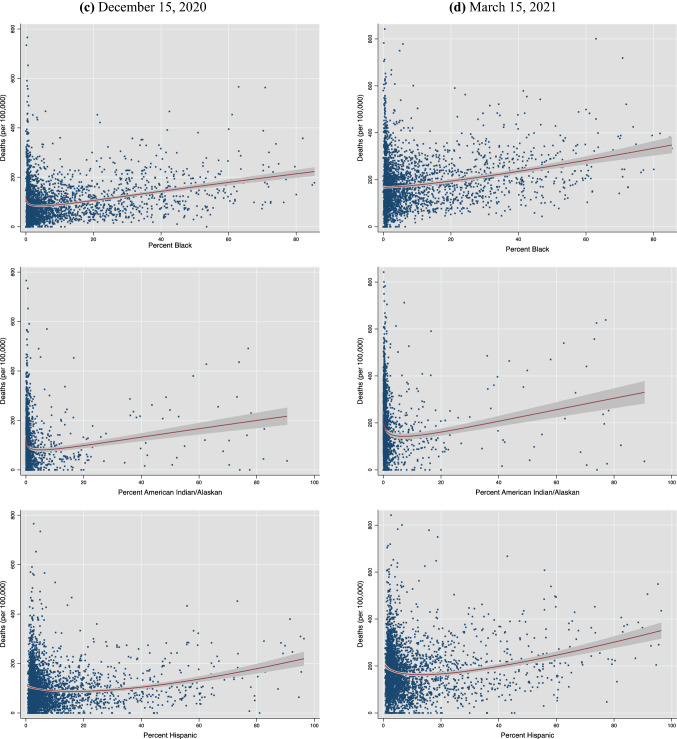
Deaths and share of racial/ethnic group in county.

**Fig. 3:**
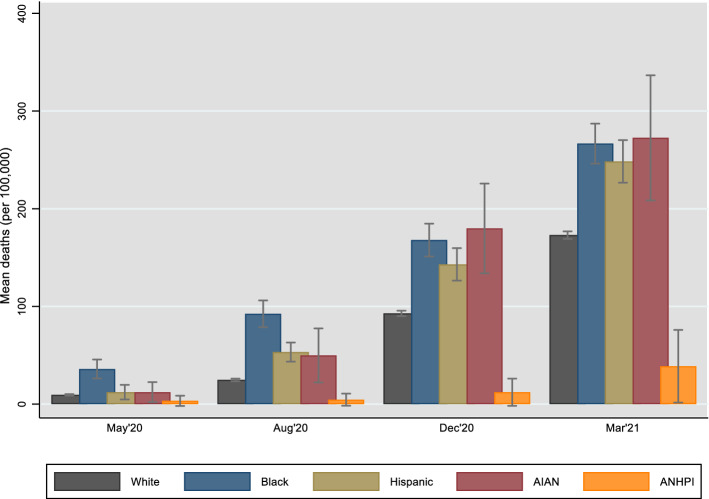
Mean COVID-19 deaths by county type based on largest racial/ethnic group

To motivate the empirical analysis, Figs. 2 and 3 show the nature and evolution of the bivariate association between county racial/ethnic composition and county COVID-19 mortality. Figure 2a–d plot, respectively, cumulative county deaths per 100,000 people on the county population share of Blacks, AIANs and Hispanics as of each of the four dates (May 15, August 15 and December 15, 2020, and March 15, 2021). A visual inspection reveals that, three months into the pandemic, only the Black percentage of a county’s population showed a positive association with county deaths. As of August 15, the bivariate relationship turned positive for Hispanics as well, while the gradient on the Black share also sharpened further. By December 2020, each of the three groups exhibited very similar plots, with a discernible positive association between their contribution to county population and total county deaths. As of March 2021, as cumulative deaths rose, the deaths vis-à-vis population share curve got steeper for all three groups.

Figure 3 documents further evidence of this trend by plotting mean deaths by type of county, where as noted counties are stratified by their largest racial/ethnic group. In the first six months of the pandemic, counties where Blacks were the plurality experienced the highest death rates—92 deaths (per 100,000 people) on average as of August 2020, nearly twice the rate of the groups with the next highest mortality (Hispanic and AIAN plurality counties). As of December 2020, however, the average death rate in AIAN plurality counties marginally surpassed that of Black counties. This remained to be the case roughly a year into the pandemic (March 15, 2021), when counties where AIANs made up the largest racial/ethnic group had an average death rate of 273, followed by Black (267), Hispanic (248) and White (173) plurality counties. Although very few in number, counties where Asians/NHPIs comprised the largest group had by far the fewest deaths (39). In sum, these simple bivariate relationships present strong baseline evidence that during the first year of the pandemic, U.S. counties that had higher shares of minority populations on average experienced higher mortality.

## Regression methodology

For the multivariate analysis, two types of regression models are adopted depending on how county racial/ethnic composition is defined and measured. In the first model, the percentage share of each racial/ethnic group in county population is used. Accordingly, the model is:

1 $$\begin{gathered} D_{i} = \beta _{0} + \beta _{1} {\text{Percent}}\_{\text{Black}}_{i} + \beta _{{\text{2}}} {\text{Percent}}\_{\text{AIAN}}_{i} {\text{ + }}\beta _{{\text{3}}} {\text{Percent}}\_{\text{Asian}}_{{\text{i}}} {\text{ + }}\beta _{{\text{4}}} {\text{Percent}}\_{\text{NHPI}}_{i} \hfill \\ \quad {\kern 1pt} {\kern 1pt} \quad {\text{ + }}\beta _{{\text{5}}} {\text{Percent}}\_{\text{Mixed}}_{i} {\text{ + }}\beta _{{\text{6}}} {\text{Percent}}\_{\text{Hispanic}}_{i} {\text{ + SEF * }}\gamma {\text{ + X * }}\Theta {\text{ + }}\varepsilon _{i} \hfill \\ \end{gathered}$$

where ${D}_{i}$ is COVID-19 cumulative deaths in county *i*, the $Percent\_$ variables represent the contribution to county population of each racial/ethnic group, $SEF$ is the vector of basic SEF with associated parameters $\gamma$, $X$ is a vector of covariates with associated parameters $\Theta$, and ${\varepsilon }_{i}$ is the regression error. The $SEF$ vector includes two income-based measures (county poverty rate and median per capita income), employment opportunities (unemployment rate) and education (ratio of population with no high school diploma). The covariates vector $X$ includes measures of population age distribution (ratio of county population 65 years and older, and 17 years and younger), health insurance coverage (uninsured rate), and county air quality (amount of fine particulate matter, PM_2.5_). Summary statistics on all the SEF and covariates are also provided in Table 1.

In this setup (model 1), an estimated race/ethnicity coefficient measures the impact on mortality of a unit (percentage point) increase in the population share of the given racial/ethnic group, all else equal, commensurate with a unit decrease in the proportion of non-Hispanic Whites. As such, the coefficient assigns an average (linear) effect for every percentage point increase in population share regardless of the level of the share. Conversely, if the size of the given racial/ethnic group has no systematic effect on COVID-19 related deaths, the estimated coefficient should be statistically indifferent from zero. A separate regression is estimated for each of the four dates during the first year of the pandemic.

In the second model, the largest group dummy/indicator variables are used to measure county racial/ethnic composition. Accordingly, the regression equation takes the form:

2 $${D}_{i}={\beta }_{0}+{\beta }_{1}{Largest\_Black}_{i}+{{\beta }_{2}Largest\_AIAN}_{i}+{{\beta }_{3}Largest\_ANHPI}_{i}+ {{\beta }_{4}Largest\_Hispanic}_{i}+SEF*\gamma +X*\Theta +{\varepsilon }_{i}$$

where all previous notations apply. Again, a separate regression is run for each of the four dates. In this regression setup (model 2), an estimated race/ethnicity coefficient measures the differential death rate in the average county where the given racial/ethnic group comprises the largest share of the population, relative to one where non-Hispanic Whites make up the largest racial group (the reference category).

Because cumulative deaths is a count variable, a negative binomial model is adopted with county population as the offset term. A negative binomial model is preferred due to the overdispersion in the cumulative deaths distribution. Robust standard errors with clustering at the state level are employed, the latter to allow for correlated errors between counties in the same state (e.g. due to state-level policy responses). All regressors are entered in standardized form. To facilitate interpretation of estimated impact, incidence rate ratios (IRRs, or exponentiated coefficients) are reported. An IRR measures the effect on death rate as a multiplicative factor.

## Results

### Racial/Ethnic composition measured by share of county population

The first set of results is from the regressions that use the percentage contribution to county population of each racial/ethnic group (model 1). These are presented in Table 2. Panel A contains the results for the first two dates (May 15 and August 15, 2020), while panel B contains the results for the latter two dates (December 15, 2020 and March 15, 2021). For each date, results from four specifications are reported. The first specification enters the racial/ethnic variables only. This gives an initial (bivariate) estimate of the race/ethnicity effect in COVID-19 mortality with respect to each racial/ethnic group. Specification (2) adds the model covariates (age, uninsured rate and pollution) and the basic SEF. Specification (3) adds state fixed effects, the latter to account for any state-level heterogeneities. In the last specification, the five counties of New York City are excluded from the analysis due to extraordinary outbreak the city experienced early in the pandemic, and is used to assess the robustness of the results to excluding potential outliers (otherwise the specification includes the full set of regressors from specification 3).^2^

**Table 2:** Covid-19 deaths and share of racial/ethnic group in county

Panel A								
	May 15, 2020				August 15, 2020			
	1	2	3	4	1	2	3	4
Black	1.572***	1.639***	1.619***	1.618***	1.672***	1.574***	1.408***	1.407***
	(5.697)	(5.052)	(7.729)	(7.748)	(10.21)	(7.635)	(10.96)	(10.96)
AIAN	1.136*	1.451***	1.441***	1.438***	1.172***	1.436***	1.416***	1.415***
	(1.743)	(4.249)	(3.752)	(3.741)	(3.274)	(6.176)	(5.506)	(5.500)
Asian	1.676***	1.158*	1.166***	1.157***	1.231***	1.066	1.061*	1.052
	(4.200)	(1.737)	(3.367)	(3.064)	(2.794)	(0.990)	(1.708)	(1.388)
NHPI	0.614***	0.446***	0.843	0.845	0.879	1.137	1.353***	1.358***
	(−2.592)	(−4.772)	(−1.323)	(−1.297)	(−1.222)	(0.964)	(3.161)	(3.226)
Mixed	0.818*	0.832*	0.725***	0.727***	0.772***	0.771***	0.810***	0.812***
	(− 1.803)	(− 1.716)	(− 3.828)	(− 3.802)	(− 2.971)	(− 3.793)	(−4.033)	(− 4.002)
Hispanic	0.967	1.089	1.085	1.080	1.257***	1.310***	1.251***	1.250***
	(− 0.304)	(0.715)	(0.744)	(0.696)	(5.237)	(4.618)	(4.507)	(4.482)
Poverty		1.035	1.020	1.021		1.010	1.044	1.048
		(0.341)	(0.245)	(0.266)		(0.144)	(0.716)	(0.767)
Unemp. Rate		0.969	0.878***	0.878***		0.987	0.922**	0.923**
		(− 0.501)	(− 2.643)	(− 2.631)		(− 0.298)	(− 2.269)	(− 2.244)
Per Capita Inc		1.509***	1.289***	1.300***		1.286***	1.161***	1.169***
		(4.015)	(3.401)	(3.303)		(3.605)	(2.896)	(2.872)
No HS Diploma		1.273**	1.285***	1.288***		1.224***	1.246***	1.248***
		(2.285)	(3.919)	(3.917)		(3.069)	(5.035)	(4.994)
Age ≥ 65		0.924	1.016	1.012		1.059	1.091**	1.088**
		(− 1.299)	(0.263)	(0.201)		(1.206)	(2.210)	(2.189)
Age ≤ 17		0.944	0.971	0.969		1.017	1.053	1.052
		(− 0.686)	(− 0.380)	(− 0.406)		(0.350)	(1.268)	(1.233)
Uninsured Rate		0.827*	0.930	0.932		0.945	0.927**	0.926**
		(− 1.658)	(− 0.976)	(− 0.946)		(− 0.974)	(− 2.006)	(− 2.014)
Pollution		1.109**	1.166***	1.163***		1.150***	1.159***	1.158***
		(2.382)	(3.194)	(3.117)		(6.252)	(6.466)	(6.425)
State effects	No	No	Yes	Yes	No	No	Yes	Yes
Observations	2900	2866	2866	2861	3116	3073	3073	3068

Panel B								
	December 15, 2020				March 15, 2021			
	1	2	3	4	1	2	3	4
Black	1.190***	1.211***	1.145***	1.145***	1.142***	1.110***	1.061***	1.060***
	(5.502)	(4.035)	(6.385)	(6.386)	(5.849)	(3.057)	(3.862)	(3.859)
AIAN	1.160***	1.240***	1.156***	1.152***	1.105***	1.182***	1.102***	1.099***
	(4.354)	(7.332)	(4.104)	(4.072)	(3.537)	(5.888)	(3.703)	(3.629)
Asian	0.961	1.044	1.064***	1.049***	0.931***	1.006	1.035**	1.026*
	(−1.123)	(1.024)	(2.611)	(2.602)	(−2.699)	(0.284)	(2.574)	(1.869)
NHPI	1.147***	0.839*	1.068	1.074	1.094**	0.838**	1.021	1.025
	(4.070)	(−1.751)	(1.259)	(1.383)	(2.285)	(−2.405)	(0.582)	(0.698)
Mixed	0.770***	0.774***	0.846***	0.850***	0.816***	0.830***	0.879***	0.881***
	(−5.464)	(−6.119)	(−5.034)	(−4.904)	(−4.027)	(−5.197)	(−5.088)	(−4.839)
Hispanic	1.069*	1.054	1.076***	1.073***	1.065**	1.065**	1.026	1.023
	(1.831)	(1.234)	(2.653)	(2.606)	(1.986)	(2.151)	(1.196)	(1.112)
Poverty		1.020	1.022	1.019		0.990	1.002	1.000
		(0.426)	(0.609)	(0.520)		(−0.320)	(0.081)	(−0.006)
Unemp. Rate		0.882***	0.936***	0.937***		0.920***	0.960**	0.961**
		(−3.592)	(−3.378)	(−3.340)		(−2.976)	(−2.375)	(−2.352)
Per Capita Inc		0.949	0.902***	0.898***		0.912**	0.874***	0.871***
		(−0.986)	(−3.446)	(−3.626)		(−2.526)	(−5.949)	(−6.155)
No HS Diploma		1.087	1.147***	1.139***		1.075*	1.107***	1.102***
		(1.366)	(4.840)	(4.858)		(1.653)	(4.715)	(4.694)
Age ≥ 65		1.209***	1.203***	1.199***		1.205***	1.189***	1.186***
		(5.332)	(6.448)	(6.661)		(6.711)	(8.548)	(8.716)
Age ≤ 17		1.168***	1.121***	1.126***		1.108***	1.095***	1.098***
		(4.439)	(6.045)	(6.038)		(3.489)	(6.264)	(6.250)
Uninsured Rate		0.960	0.924***	0.924***		0.989	0.942***	0.942***
		(−0.868)	(−3.629)	(−3.644)		(−0.261)	(−2.915)	(−2.914)
Pollution		1.026	1.047*	1.043*		1.056***	1.048**	1.045**
		(1.081)	(1.859)	(1.791)		(3.061)	(2.064)	(1.961)
State effects	No	No	Yes	Yes	No	No	Yes	Yes
Observations	3136	3091	3091	3086	3136	3091	3091	3086

Incidence rate ratios (IRR) are reported. t-statistics based on robust standard errors clustered at the state level are shown in parentheses. Regressors are entered in standardized form and each regression also included a constant. Specification (4) excludes New York City. ***p < 0.01, **p < 0.05, *p < 0.1

The first half of panel A presents the results for the earliest date in this study—May 15, 2020, roughly three months into the pandemic. Because IRRs are reported, an estimate greater than one shows a positive (increasing) effect and an estimate less than one shows a negative (decreasing) effect. Seen across all specifications, the results generally indicate that early in the pandemic, counties with a larger share of Black, AIAN and Asian populations experienced higher mortality due to COVID-19. In contrast, an increase in the share of the mixed (two or more races) population was associated with decreased mortality, as did the share of NHPI (when statistically significant). Statistically, the share of Hispanics had no impact on mortality.

When only the racial/ethnic variables are included as regressors (specification 1), a 1- standard deviation (14.3 percentage point) increase in the share of the Black population was associated with a 57 percent increase in mortality. A corresponding unit-standard deviation increase in the AIAN and Asian share raised mortality by 14 percent and 68 percent, respectively. The Black coefficient more or less holds steady when covariates, basic SEF and state effects are included in specifications (2) and (3). However, interestingly, the addition of these variables has noticeably contrasting effects on the AIAN and Asian estimates—the former effectively triples (to 44 percent in the full specification, 3) while the latter decreases by three-quarters (to just 17 percent). Once the full set of potential confounders are controlled for, however, excluding potentially outlier counties does not seem to affect the estimated racial/ethnic effects much (specification 4). In sum, the results show that at the beginning of the pandemic, the estimated race/ethnicity effect in COVID-19 mortality was highest for Blacks and AIANs. Furthermore, whereas the Asian effect was also large and significant, basic covariates and SEF collectively mediate about 75 percent of the effect. Geography may have played a role in the higher mortality of Asians during the early months, as the disease took a heavy toll in areas where there are large concentrations of Asian populations (U.S. Northeast, such as New York, New Jersey and Massachusetts).^3^

Roughly six months into the pandemic, the race/ethnicity effect for the various groups has already evolved some. The Black effect starts out high (67 percent) but, once accounting for all potential confounders, declines to 41 percent, an effect that is comparable to that for AIANs. The Asian effect had decreased substantially—based on the full/extended specification, a unit standard deviation (2.9 percentage point) increase in the Asian share of the county population was associated with only a 6 percent rise in mortality (p < 0.1). The share of a county’s population with Hispanic ethnicity, however, was by then associated with an elevated county death rate. Higher share of the mixed population continued to be a predictor of lower county-level COVID-19 mortality.

As of December 15, 2020 (panel B), only the Black and AIAN shares of the population were consistently associated with raised mortality. Accounting for county variables and state effects (specification 3), a unit-standard deviation increase in the Black share was, on average, associated with a 15 percent increase in county death rate, virtually the same as the corresponding estimate for AIANs. The mixed share again consistently indicated decreased mortality. Interestingly, the Asian and Hispanic shares were associated with increased mortality (p < 0.01) only in the full specification (3) and sensitivity check (4). Their estimated coefficients were also somewhat comparable (6.4–7.6 percent, respectively).

As the pandemic proceeded through various waves and enveloped the country, racial/ethnic composition, perhaps unsurprisingly, became less of a factor in county-level variation in mortality. Still, as of March 15, 2021, a year into the pandemic, Black and AIAN population shares remained statistically significant predictors, even after accounting for basic SEF and confounders. A 1-standard deviation (7.3 percentage point) increase in the share of AIANs in a county increased mortality by 10 percent. A 1-standard deviation increase in the Black population raised mortality by 6 percent. The corresponding effect for the Asian share was 3.5 percent, but the estimate was only sporadically statistically significant across specifications, while the mixed share of the population continued to be consistently predictive of decreased mortality. The Hispanic effect was statistically insignificant in the full specification.

Finally, examining the estimates on the covariates and basic SEF, the share of the older population (65 years and over) as expected was associated with higher mortality in a majority of specifications. The share of the youngest population (17 and younger) also indicated a higher risk of county mortality in the latter two dates. The ratio of the uninsured population, whenever significant, was mildly associated with lower county mortality. Poor air quality, on the other hand, consistently predicted increased mortality. Among the basic SEF, lower education was the most potent predictor of higher county COVID-19 mortality. A unit-standard deviation (6.3 percentage point) increase in the share of a county’s population with no high school diploma was associated with about a 26 percent increase in mortality during the first six months of the pandemic. The effect was about half in size, on average, for the latter two dates, but it remained statistically highly significant. The result is likely signifying the role of occupation, whereby the less educated populace is systematically overrepresented in frontline and essential jobs, which carry higher risk of infection and mortality from COVID-19.

A county’s per capita income was a positive predictor of deaths as of the first two milestones, but by December 2020 and March 2021 the estimated coefficient has switched signs and higher per capita income was associated with lower mortality. The pattern of results may simply be an artifact of the pattern in the geographical spread of the disease during the course of the year, from the relatively higher income Northeastern and Western United States early on to the rest of the country in later months. For the most part, higher county unemployment was also associated with slightly reduced mortality.

### Racial/Ethnic composition proxied by a county’s largest racial/ethnic group

The second set of results, reported in Table 3, employs dummy/indicator variables on the largest racial/ethnic group to proxy county racial/ethnic composition (model 2). Again, results are reported for the four dates that are analyzed in this study and the table presents results from multiple specifications that, beginning with just the racial/ethnic variables, incrementally add basic covariates and SEF, and state effects. The last specification excludes New York City. White plurality counties form the reference category for the racial/ethnic estimates.

**Table 3:** Covid-19 deaths and largest racial/ethnic group in county

Panel A								
	May 15, 2020				August 15, 2020			
	1	2	3	4	1	2	3	4
Black	3.547***	2.690***	2.307***	2.325***	3.521***	1.888***	1.561***	1.565***
	(6.165)	(4.962)	(5.745)	(5.837)	(13.28)	(3.980)	(4.975)	(4.992)
AIAN	1.461	5.361***	6.227***	6.036***	2.070*	3.983***	5.578***	5.521***
	(0.748)	(3.242)	(3.169)	(3.157)	(1.869)	(3.566)	(3.764)	(3.764)
ANHPI	0.340*	0.123***	1.153	1.102	0.240***	0.190***	0.714***	0.708***
	(−1.881)	(−6.039)	(1.019)	(0.625)	(−2.868)	(−6.567)	(−3.280)	(−3.154)
Hispanic	1.329	0.833	0.934	0.845	2.063***	1.157	1.147	1.113
	(0.589)	(−0.548)	(−0.332)	(−0.844)	(4.521)	(0.756)	(1.084)	(0.790)
Poverty		1.357**	1.218**	1.217**		1.236**	1.169**	1.170**
		(2.398)	(2.117)	(2.093)		(2.533)	(2.294)	(2.289)
Unemp. Rate		1.040	0.924	0.924		1.076	0.964	0.965
		(0.597)	(−1.288)	(−1.281)		(1.223)	(−0.817)	(−0.797)
Per Capita Inc		1.896***	1.463***	1.469***		1.544***	1.250***	1.252***
		(4.993)	(4.520)	(4.428)		(5.068)	(4.001)	(3.912)
No HS Diploma		1.484***	1.442***	1.440***		1.489***	1.399***	1.398***
		(3.410)	(5.167)	(5.147)		(4.859)	(6.163)	(6.088)
Age ≥ 65		0.838***	0.953	0.952		0.958	1.016	1.015
		(−2.763)	(−0.724)	(−0.753)		(−1.018)	(0.401)	(0.383)
Age ≤ 17		0.889	0.961	0.966		1.017	1.077**	1.079**
		(−1.509)	(−0.624)	(−0.531)		(0.336)	(2.138)	(2.192)
Uninsured Rate		0.896	0.932	0.930		1.050	0.959	0.957
		(−0.813)	(−0.808)	(−0.834)		(0.541)	(−0.749)	(−0.788)
Pollution		1.155***	1.229***	1.216***		1.158***	1.196***	1.190***
		(3.804)	(4.185)	(4.149)		(6.471)	(6.661)	(6.902)
State effects	No	No	Yes	Yes	No	No	Yes	Yes
Observations	2,900	2,866	2,866	2,861	3,116	3,073	3,073	3,068

Panel B								
	December 15, 2020				March 15, 2021			
	1	2	3	4	1	2	3	4
Black	1.815***	1.554***	1.231***	1.240***	1.538***	1.272***	1.101**	1.106***
	(9.589)	(3.817)	(4.045)	(4.233)	(7.784)	(2.892)	(2.505)	(2.647)
AIAN	1.968***	2.419***	2.061***	2.020***	1.598**	2.234***	1.750***	1.726***
	(3.034)	(3.681)	(2.636)	(2.588)	(2.139)	(3.984)	(2.838)	(2.777)
ANHPI	0.209***	0.448***	1.190***	1.201***	0.289***	0.769**	1.422***	1.433***
	(−4.834)	(−4.563)	(3.037)	(3.254)	(−3.044)	(−2.110)	(11.22)	(12.67)
Hispanic	1.535**	1.166	1.198***	1.165**	1.430***	1.170*	1.084	1.068
	(2.505)	(1.021)	(2.637)	(1.983)	(3.052)	(1.901)	(1.603)	(1.250)
Poverty		1.091*	1.067*	1.059		1.018	1.019	1.014
		(1.782)	(1.675)	(1.500)		(0.477)	(0.688)	(0.514)
Unemp. Rate		0.900***	0.946***	0.947**		0.929**	0.962**	0.962**
		(−2.648)	(−2.592)	(−2.554)		(−2.414)	(−2.325)	(−2.314)
Per Capita Inc		0.994	0.938*	0.927**		0.928*	0.889***	0.881***
		(−0.104)	(−1.724)	(−2.384)		(−1.704)	(−4.670)	(−6.023)
No HS Diploma		1.156**	1.199***	1.188***		1.132***	1.141***	1.133***
		(2.466)	(5.847)	(6.189)		(2.850)	(6.314)	(6.606)
Age ≥ 65		1.200***	1.175***	1.175***		1.213***	1.184***	1.184***
		(6.708)	(6.663)	(6.681)		(8.276)	(9.479)	(9.516)
Age ≤ 17		1.177***	1.125***	1.134***		1.124***	1.098***	1.103***
		(4.108)	(6.050)	(6.064)		(3.565)	(6.199)	(6.206)
Uninsured Rate		0.974	0.931***	0.929***		1.002	0.943***	0.942***
		(−0.400)	(−2.848)	(−2.962)		(0.0335)	(−2.653)	(−2.704)
Pollution		1.035	1.058**	1.050**		1.056***	1.049**	1.044*
		(1.493)	(2.015)	(1.977)		(3.046)	(2.041)	(1.910)
State effects	No	No	Yes	Yes	No	No	Yes	Yes
Observations	3,136	3,091	3,091	3,086	3,136	3,091	3,091	3,086

Incidence rate ratios (IRR) are reported. t-statistics based on robust standard errors clustered at the state level are shown in parentheses. ANHPI equals 1 if a county’s largest racial/ethnic group is Asian or NHPI, zero otherwise. Regressors (except racial/ethnic indicators) are entered in standardized form and each regression also included a constant. Specification (4) excludes New York City. ***p < 0.01, **p < 0.05, *p < 0.1

According to the results in panel A, three months into the COVID-19 pandemic, counties in which Blacks or AIANs comprise the largest racial/ethnic group had considerably higher mortality. The estimated race/ethnicity effect for these groups is quite high. In the full specification (3) that accounts for covariates, basic SEF and state effects, a typical county that has Blacks as the largest racial group had 2.3 times the mortality rate of a White plurality county (or, put differently, a 130 percent increase in mortality). The corresponding estimate for an AIAN county is even higher – a death rate that is 523 percent higher. Similar to the earlier results, incrementally accounting for potential confounders is found to be consequential, albeit in contrasting patterns for the two groups. The Black estimate is about a third smaller in the extended specification (3) than in the simplest (bivariate) specification (1). The AIAN estimate, however, does not even turn statistically significant until confounding variables are included in the regressions. These results underscore the importance of properly accounting for sociodemographic and related factors when assessing racial/ethnic disparities in COVID-19 mortality. Similar to the results that were based on county population shares, as of May 2020, being a county where Hispanics make up the largest racial/ethnic group was not associated with a statistically significant difference in the death rate.

Six months into the pandemic, mortality outcomes by race/ethnicity remained qualitatively similar. Counties where Blacks or AIANs are the largest group had disproportionately higher deaths than White plurality counties, while those where Asians/NHPIs make up the largest group had lower mortality. Relative to earlier in the pandemic, however, the Black estimate declined considerably (by about a third) while the AIAN estimate remained quite elevated. As of August 2020, the typical county in which AIANs make up the largest racial group experienced a death rate that was 460 percent higher than one in which Whites are the plurality. By comparison, Asian/NHPI counties on average experienced mortality rates that were on average about 30 percent lower. Again, it is worth noting that the estimated effects for Black and AIAN show quite contrasting responses to the inclusion of covariates and basic SEF. Specifically, it appears that failure to account for potential confounders leads to an upward bias in the Black estimate while it has the opposite effect on the AIAN estimate. Simple bivariate analyses therefore can be misleading when assessing the impact of race/ethnicity on COVID-19 mortality.

By December 2020, the race effect remained statistically significant for the two groups (Blacks and AIANs) but its size was much smaller (panel B). Relative to the average county where Whites make up the largest group, mortality was about 23 percent and 106 percent higher in the typical county in which Blacks and AIANs form the largest group, respectively (based on the estimates in the full specification, 3). A county in which Hispanics make up the plurality also experienced a higher death rate (about 20 percent higher). The race/ethnicity effect waned even further a year into the pandemic, as of March 15, 2021. Even so, a county in which AIANs are the plurality had deaths that were 1.75 times higher. The corresponding effect for Black plurality counties was 1.1. Notably, Asian/NHPI estimates showed elevated mortality, albeit only in the full/extended specification (3) and sensitivity check (4) and as of the last two dates (December 2020 and March 2021).

Again looking at the impact of basic covariates and SEF, counties with higher shares of older as well as younger populations experienced higher mortality rates, particularly in the latter half of the first year of the pandemic. In this model, higher poverty, all else equal, predicted higher mortality during most of the year. Conversely, higher unemployment and uninsured rate was each associated with a small but statistically significant decrease in mortality in the second half of the year (December 2020 and March 2021). Richer counties (by per capita income) experienced more deaths early on in the pandemic, an effect that is reversed later in the year. Lower education, however, again consistently and potently predicted higher mortality across all four dates.

### Summary of regression results

**Fig. 4:**
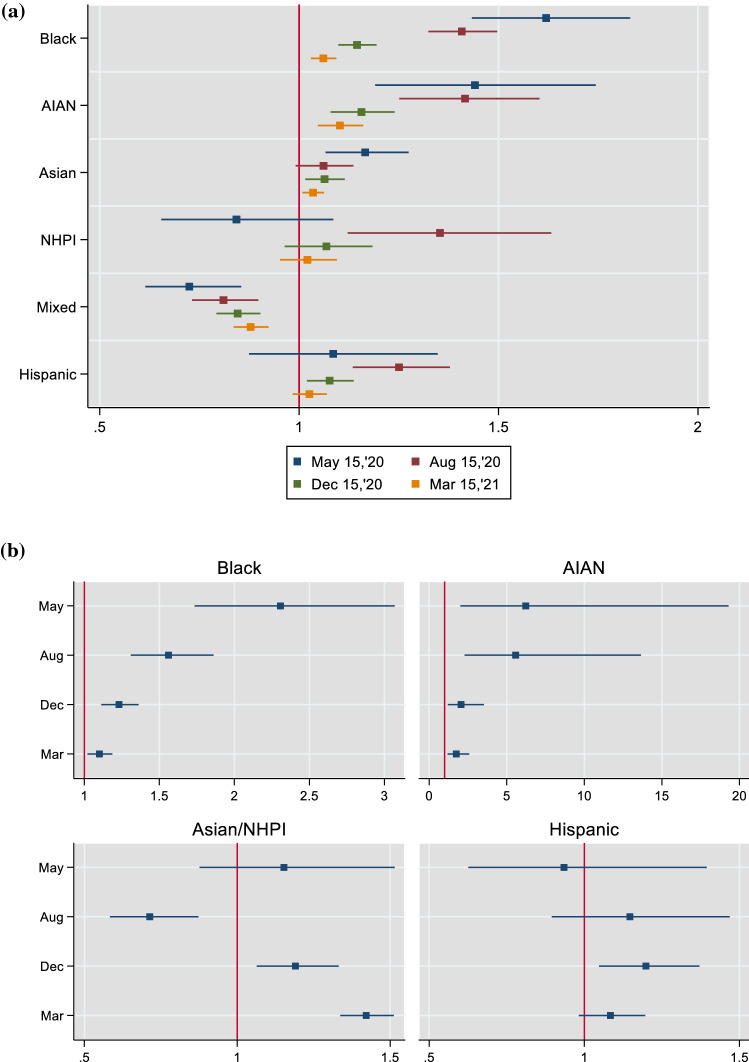
**a** The race/ethnicity effect during the first year of the pandemic—estimated IRR on share of county population. Incidence rate ratios (IRR) and 95% confidence intervals are shown. **b** The race/ethnicity effect during the first year of the pandemic – estimated IRR on largest racial/ethnic group dummy. Incidence rate ratios (IRR) and 95% confidence intervals are shown. The dates are May 15, August 15, and December 15, 2020; and March 15, 2021

To summarize, Fig. 4a collates the estimated race/ethnicity coefficients (and confidence intervals) across the four dates from the regressions that are based on population shares (Table 2). The coefficients are from the full/extended specification (specification 3). The figure visually conveys the two important trends that are highlighted earlier. First, for all groups, the race/ethnicity effect—as measured by the impact on county-level mortality of a group’s share of county population—waned as time elapsed during the first year of the pandemic. Second, the effect remained highly statistically significant (p < 0.01) across all four dates only for three groups (Black, AIAN and mixed). Black and AIAN were associated with elevated mortality. In contrast, a higher share of people from two or more races (mixed) in a county’s population was consistently associated with reduced mortality.

Figure 4b also collects the estimated coefficients on the largest racial/ethnic group dummies for all four dates. These are again based on the full/extended specification (specification 3 in Table 3). Observing the pattern over time, again only the coefficients on Black and AIAN reveal clearer trends. Both decreased during the course of the year, although they remained statistically significant throughout. The AIAN estimate, however, was considerably larger, especially as of the first two dates (notice the different scales for the two variables) albeit with much larger confidence intervals. As for the other two groups, the Hispanic effect was statistically indifferent from zero for most of the year once accounting for basic SEF, while the Asian/NHPI coefficient oscillated between positive and negative values.

## Conclusions

This study revisited the important issue of disparities in COVID-19 outcomes in the United States along racial and ethnic lines. Focusing on mortality during the first year of the pandemic, its aim was to: (1) provide a more complete picture of racial and ethnic disparities and their evolution; and (2) examine the extent to which the disparities could be explained by existing differences in basic socioeconomic factors. Because many of the extant studies on the topic took place relatively early during the pandemic and mostly gave a snapshot picture of disparities, a retrospective, longitudinal investigation of the kind performed in this study is valuable.

The analysis adopted an ecological regression framework and used county-level data. Regressions gauging the impact of county racial/ethnic composition on county mortality were performed as of four dates during the first 13 months of the pandemic. All major racial/ethnic classifications as identified by the U.S. Census Bureau were considered and county racial composition was flexibly defined in two ways—by each group’s contribution to county population and by indicator variables of group plurality.

Bivariate plots indicated a positive association between county COVID-19 mortality and the size of Black, Hispanic and AIAN residents. Yet, results from the multivariate ecological regressions revealed two main trends. First, when accounting for basic covariates and socioeconomic factors, the race/ethnicity effect behaved in notably different ways for the three groups. The Hispanic effect was often close to fully mediated (that is, became statistically insignificant). The Black effect often decreased—by 4–56 percent, depending on the date of analysis and specification, especially in the model that uses group plurality as a measure of racial composition—but always remained statistically significant. The AIAN effect, however, was either largely unchanged (in the specifications that used contribution to county populations) or increased (in the specifications that used group plurality). Second, for all three groups the race/ethnicity-mortality association generally waned in magnitude during the latter months of the first year of the pandemic.

On a basic level, the question is “what is the likely cause of differences in the COVID-19 mortality risk along racial/ethnic lines?” Absent genetic or biological explanations, the answer broadly rests with the legacy and ongoing effects of structural racism—the multi-faceted ways in which racial discrimination causes inferior health outcomes in minority communities [4, 23, 28, 35]. Many of the effects of structural racism on health operate indirectly through social, economic and institutional channels, such as education, employment, housing, healthcare and the justice system. But there are direct channels as well, for instance when perceptions and experiences of discrimination become health stressors themselves or worsen the impact of environmental stressors [28, 36]. Regardless, among the main effects on health is the preponderance of various comorbidities in these communities, which has made them particularly susceptible to the worst effects of the COVID-19 pandemic.

Viewed through this lens, the almost total mediation of the Hispanic effect on mortality by basic socioeconomic factors is notable. Policy-wise, it suggests that, for Hispanic populations, addressing basic inequities in education, employment and income can be effective in preventing similarly worse outcomes in a future health crisis (and also perhaps in remedying inferior health outcomes generally). For Black and AIAN populations, however, the fact that an unexplained, statistically significant race effect remains after accounting for basic socioeconomic factors means that tackling other channels and manifestations of structural racism in health is necessary.

For Black Americans, these other channels have been identified and acknowledged in the context of COVID-19. In healthcare, they include barriers to access, inferior quality care and low utilization [36, 37]. For example, higher mortality has been shown to be positively associated with lack of internet access, an important means of accessing up-to-date health and safety information, not to mention an indispensable tool for learning and working remotely [19, 38]. Medical mistrust—a result of the community’s history with healthcare discrimination, scientific racism, and everyday discrimination, perceived and actual—is associated with reluctance to access care, decreased engagement in safety and protective practices, and elevated tendency to involve in conspiracy theories and beliefs [24, 35, 39, 40]. African Americans, for instance, had significantly lower COVID-19 vaccination rates, particularly in the early phases of the vaccination campaign [41, 42]. More broadly, social, community and institutional structures have been shown to raise the mortality risk for the Black community. These include, among others, concentrated deprivation [23] and residential segregation [4].

Historically, AIAN communities have also long endured discrimination [43, 44]. And, although the evidence pertaining to their specific experiences and the mechanisms that have led to their worse outcomes during COVID-19 is more limited, some of the very same channels that are highlighted above that afflict Black communities are also likely to apply to them. For example, in surveys conducted during the pandemic, AIANs reported comparable levels of medical mistrust as Blacks [24]. The results in this paper underscore that in the discussion of the pandemic’s effect on minority communities, AIANs should also be at the forefront. They have borne a disproportionate share of the mortality burden and, given that their higher mortality risk does not appear to be mediated to a great extent by socioeconomic conditions, a targeted policy response, grounded in scientific investigation and evidence, would be necessary to prevent similarly devastating outcomes for the community in the event of a future pandemic [45].

Even so, for both Black and AIAN communities, the overriding implication of the results in this study is that, in order for a policy response to effectively address the observed disparities, it needs a multi-pronged approach and a long-term commitment. It must confront many of the broader structural inequities in the social and economic arenas, including in education, employment, housing and healthcare, as well as disparities in wealth and power that have resulted in entrenched inequalities in health outcomes. But it should also be attuned to the specific cultural and historical sensitivities that have caused these communities to access and utilize healthcare at sub-optimal rates [27, 44, 45]. As such, the policy response requires careful investigation, planning, building societal awareness and consensus and, inevitably, significant mobilization of resources. Only through such a committed and deliberate approach would we be able, as a society, to eliminate racial and ethnic health inequities in the long run.

Certain limitations and caveats apply to the analyses and results in this study. First, given that county-level data and an ecological regression framework are used, direct inferences cannot be drawn about the association between race/ethnicity and COVID-19 outcomes at the individual level. As noted in the introduction, ideally the kind of analyses conducted in this study are best performed with person-level data on COVID-19 outcomes, race/ethnicity and other requisite covariates, and with the necessary adjustments for age. But such data are not reported at scale in the U.S. Second, county death data may suffer from some measurement error due to, among others, inconsistencies in definition and reporting standards. Third, given the inherently complex nature of the issue at hand, the estimated race/ethnicity coefficients are measuring simple, reduced-form associations, not causal effects (in the statistical sense). Typically, the issue is further complicated by questions surrounding covariate choice, model selection and other potential sources of confounding. For instance, to the extent that the prevalence and impact of comorbidities is not adequately captured by basic SEF and environmental variables, inclusion of community disease proxies as additional covariates would be warranted. Such measures are available [19] and their inclusion can perhaps further mediate the race/ethnicity effect by capturing direct effects on mortality. Similarly, in future work, a more flexible regression model can be employed to investigate potential nonlinearities in the race/ethnicity effect, including interaction effects between the various racial/ethnic compositions. Despite these limitations, the comprehensive nature of the study—in its analysis of the race/ethnicity effect for all minority groups and over time, its flexible measurement of county racial/ethnic composition, and its internal consistency in measurement and estimation approaches—should make for a valuable contribution and an instructive resource for policymaking.

## Supplementary Information

Below is the link to the electronic supplementary material.

**Additional file 1**.

## Data Availability

The study used publicly available data. The data can be made available upon request from the corresponding author.

## Footnotes

1 It should be noted that the linking of the first two dates to COVID-19 waves in the U.S. is an approximation [30]. In fact, the criteria and approaches for dating waves and surge behavior are varied and complex [31, 32]. The U.S. declared a national emergency on March 13, 2020 [33] and the first vaccine was given to a member of the public on December 14 [34].

2 In a full/extended set of results for each date, there was also an intermediate specification that adds just the model covariates only (without the basic SEF) and another robustness test that excluded zero-death counties. Those specifications are not reported here for brevity. The full sets of results that contain all six specifications for each date (and which can be used to roughly gauge how much of the race/ethnicity effect is being mediated in successive steps) are put together in the online appendix.

3 For instance, of the 100 counties with the highest death rates as of May 15, 2020, a third (34 counties) were in New York, New Jersey, Massachusetts and Connecticut. The Asian share of the population in those counties was 8.4 percent, 5.5 times the full sample average.
