# Supplementary material for: Race, ethnicity and mortality in the United States during the first year of the COVID-19 pandemic: an assessment
Source: Discov Soc Sci Health. 2022 Sep 26;2(1):16. doi: 10.1007/s44155-022-00019-9 (PMC9511463; doi:10.1007/s44155-022-00019-9)
Supplement: Supplementary file 1 — Additional file 1. [file 44155_2022_19_MOESM1_ESM.docx]

**Additional file 1: Full/extended set of regressions by analysis date**

**Table 1:** Covid-19 deaths and share of racial/ethnic group in county – As of May 15, 2020

|  | 1 | 2 | 3 | 4 | 5 | 6 |
| --- | --- | --- | --- | --- | --- | --- |
| Black | 1.572*** | 1.572*** | 1.639*** | 1.619*** | 1.618*** | 1.453*** |
|  | (5.697) | (4.668) | (5.052) | (7.729) | (7.748) | (6.388) |
| AIAN | 1.136* | 1.369*** | 1.451*** | 1.441*** | 1.438*** | 1.539*** |
|  | (1.743) | (4.089) | (4.249) | (3.752) | (3.741) | (6.856) |
| Asian | 1.676*** | 1.395*** | 1.158* | 1.166*** | 1.157*** | 1.175*** |
|  | (4.200) | (3.515) | (1.737) | (3.367) | (3.064) | (5.515) |
| NHPI | 0.614*** | 0.386*** | 0.446*** | 0.843 | 0.845 | 0.733** |
|  | (-2.592) | (-5.202) | (-4.772) | (-1.323) | (-1.297) | (-2.108) |
| Mixed | 0.818* | 0.830* | 0.832* | 0.725*** | 0.727*** | 0.695*** |
|  | (-1.803) | (-1.760) | (-1.716) | (-3.828) | (-3.802) | (-5.194) |
| Hispanic | 0.967 | 1.162 | 1.089 | 1.085 | 1.080 | 1.042 |
|  | (-0.304) | (1.175) | (0.715) | (0.744) | (0.696) | (0.415) |
| Poverty |  |  | 1.035 | 1.020 | 1.021 | 1.046 |
|  |  |  | (0.341) | (0.245) | (0.266) | (0.624) |
| Unemp. Rate |  |  | 0.969 | 0.878*** | 0.878*** | 0.916* |
|  |  |  | (-0.501) | (-2.643) | (-2.631) | (-1.714) |
| Per Capita Inc. |  |  | 1.509*** | 1.289*** | 1.300*** | 1.175*** |
|  |  |  | (4.015) | (3.401) | (3.303) | (2.753) |
| No HS Diploma |  |  | 1.273** | 1.285*** | 1.288*** | 1.369*** |
|  |  |  | (2.285) | (3.919) | (3.917) | (4.342) |
| Age ≥ 65 |  | 0.952 | 0.924 | 1.016 | 1.012 | 1.214*** |
|  |  | (-0.656) | (-1.299) | (0.263) | (0.201) | (3.941) |
| Age ≤ 17 |  | 0.969 | 0.944 | 0.971 | 0.969 | 1.004 |
|  |  | (-0.338) | (-0.686) | (-0.380) | (-0.406) | (0.0716) |
| Uninsured Rate |  | 0.785** | 0.827* | 0.930 | 0.932 | 0.915 |
|  |  | (-2.489) | (-1.658) | (-0.976) | (-0.946) | (-1.140) |
| Pollution |  | 1.116** | 1.109** | 1.166*** | 1.163*** | 1.065 |
|  |  | (2.503) | (2.382) | (3.194) | (3.117) | (1.551) |
| alpha | 2.004*** | 1.878*** | 1.804*** | 1.248*** | 1.253*** | 0.655*** |
|  | (10.62) | (10.27) | (10.05) | (2.910) | (2.959) | (-7.695) |
| State effects | No | No | No | Yes | Yes | Yes |
| Observations | 2,900 | 2,866 | 2,866 | 2,866 | 2,861 | 1,613 |

*Notes*: Incidence rate ratios (IRR) are reported. t-statistics based on robust standard errors clustered at the state level are shown in parentheses. Regressors are entered in standardized form and each regression also included a constant. Specification (5) excludes New York City. Specification (6) excludes zero-death counties. *** p<0.01, ** p<0.05, * p<0.1.

**Table 2:** Covid-19 deaths and share of racial/ethnic group in county – As of August 15, 2020

|  | 1 | 2 | 3 | 4 | 5 | 6 |
| --- | --- | --- | --- | --- | --- | --- |
| Black | 1.672*** | 1.543*** | 1.574*** | 1.408*** | 1.407*** | 1.379*** |
|  | (10.21) | (8.538) | (7.635) | (10.96) | (10.96) | (10.37) |
| AIAN | 1.172*** | 1.383*** | 1.436*** | 1.416*** | 1.415*** | 1.450*** |
|  | (3.274) | (5.736) | (6.176) | (5.506) | (5.500) | (6.299) |
| Asian | 1.231*** | 1.174** | 1.066 | 1.061* | 1.052 | 1.102*** |
|  | (2.794) | (2.350) | (0.990) | (1.708) | (1.388) | (2.607) |
| NHPI | 0.879 | 1.052 | 1.137 | 1.353*** | 1.358*** | 1.169* |
|  | (-1.222) | (0.346) | (0.964) | (3.161) | (3.226) | (1.852) |
| Mixed | 0.772*** | 0.764*** | 0.771*** | 0.810*** | 0.812*** | 0.775*** |
|  | (-2.971) | (-3.877) | (-3.793) | (-4.033) | (-4.002) | (-5.774) |
| Hispanic | 1.257*** | 1.385*** | 1.310*** | 1.251*** | 1.250*** | 1.242*** |
|  | (5.237) | (5.211) | (4.618) | (4.507) | (4.482) | (4.589) |
| Poverty |  |  | 1.010 | 1.044 | 1.048 | 1.031 |
|  |  |  | (0.144) | (0.716) | (0.767) | (0.506) |
| Unemp. Rate |  |  | 0.987 | 0.922** | 0.923** | 0.928** |
|  |  |  | (-0.298) | (-2.269) | (-2.244) | (-2.244) |
| Per Capita Inc. |  |  | 1.286*** | 1.161*** | 1.169*** | 1.087* |
|  |  |  | (3.605) | (2.896) | (2.872) | (1.879) |
| No HS Diploma |  |  | 1.224*** | 1.246*** | 1.248*** | 1.232*** |
|  |  |  | (3.069) | (5.035) | (4.994) | (5.317) |
| Age ≥ 65 |  | 1.078 | 1.059 | 1.091** | 1.088** | 1.197*** |
|  |  | (1.523) | (1.206) | (2.210) | (2.189) | (5.273) |
| Age ≤ 17 |  | 1.035 | 1.017 | 1.053 | 1.052 | 1.048 |
|  |  | (0.701) | (0.350) | (1.268) | (1.233) | (1.443) |
| Uninsured Rate |  | 0.937 | 0.945 | 0.927** | 0.926** | 0.889*** |
|  |  | (-1.218) | (-0.974) | (-2.006) | (-2.014) | (-3.275) |
| Pollution |  | 1.161*** | 1.150*** | 1.159*** | 1.158*** | 1.108*** |
|  |  | (6.441) | (6.252) | (6.466) | (6.425) | (4.890) |
| alpha | 0.916 | 0.824** | 0.798** | 0.579*** | 0.580*** | 0.422*** |
|  | (-0.873) | (-2.035) | (-2.494) | (-5.387) | (-5.322) | (-11.32) |
| State effects | No | No | No | Yes | Yes | Yes |
| Observations | 3,116 | 3,073 | 3,073 | 3,073 | 3,068 | 2,355 |

*Notes*: Incidence rate ratios (IRR) are reported. t-statistics based on robust standard errors clustered at the state level are shown in parentheses. Regressors are entered in standardized form and each regression also included a constant. Specification (5) excludes New York City. Specification (6) excludes zero-death counties. *** p<0.01, ** p<0.05, * p<0.1.

**Table 3:** Covid-19 deaths and share of racial/ethnic group in county – As of December 15, 2020

|  | 1 | 2 | 3 | 4 | 5 | 6 |
| --- | --- | --- | --- | --- | --- | --- |
| Black | 1.190*** | 1.196*** | 1.211*** | 1.145*** | 1.145*** | 1.153*** |
|  | (5.502) | (5.885) | (4.035) | (6.385) | (6.386) | (6.552) |
| AIAN | 1.160*** | 1.201*** | 1.240*** | 1.156*** | 1.152*** | 1.155*** |
|  | (4.354) | (5.789) | (7.332) | (4.104) | (4.072) | (4.136) |
| Asian | 0.961 | 1.011 | 1.044 | 1.064*** | 1.049*** | 1.067*** |
|  | (-1.123) | (0.270) | (1.024) | (2.611) | (2.602) | (2.651) |
| NHPI | 1.147*** | 0.855 | 0.839* | 1.068 | 1.074 | 1.051 |
|  | (4.070) | (-1.461) | (-1.751) | (1.259) | (1.383) | (0.964) |
| Mixed | 0.770*** | 0.759*** | 0.774*** | 0.846*** | 0.850*** | 0.843*** |
|  | (-5.464) | (-5.679) | (-6.119) | (-5.034) | (-4.904) | (-5.025) |
| Hispanic | 1.069* | 1.062 | 1.054 | 1.076*** | 1.073*** | 1.081*** |
|  | (1.831) | (1.516) | (1.234) | (2.653) | (2.606) | (2.771) |
| Poverty |  |  | 1.020 | 1.022 | 1.019 | 1.019 |
|  |  |  | (0.426) | (0.609) | (0.520) | (0.519) |
| Unemp. Rate |  |  | 0.882*** | 0.936*** | 0.937*** | 0.931*** |
|  |  |  | (-3.592) | (-3.378) | (-3.340) | (-3.654) |
| Per Capita Inc. |  |  | 0.949 | 0.902*** | 0.898*** | 0.897*** |
|  |  |  | (-0.986) | (-3.446) | (-3.626) | (-3.572) |
| No HS Diploma |  |  | 1.087 | 1.147*** | 1.139*** | 1.140*** |
|  |  |  | (1.366) | (4.840) | (4.858) | (4.766) |
| Age ≥ 65 |  | 1.210*** | 1.209*** | 1.203*** | 1.199*** | 1.221*** |
|  |  | (5.124) | (5.332) | (6.448) | (6.661) | (6.672) |
| Age ≤ 17 |  | 1.182*** | 1.168*** | 1.121*** | 1.126*** | 1.120*** |
|  |  | (4.411) | (4.439) | (6.045) | (6.038) | (5.834) |
| Uninsured Rate |  | 1.011 | 0.960 | 0.924*** | 0.924*** | 0.924*** |
|  |  | (0.318) | (-0.868) | (-3.629) | (-3.644) | (-3.987) |
| Pollution |  | 1.025 | 1.026 | 1.047* | 1.043* | 1.040 |
|  |  | (1.048) | (1.081) | (1.859) | (1.791) | (1.592) |
| alpha | 0.384*** | 0.350*** | 0.340*** | 0.223*** | 0.221*** | 0.209*** |
|  | (-9.275) | (-10.17) | (-9.910) | (-14.83) | (-14.93) | (-17.15) |
| State effects | No | No | No | Yes | Yes | Yes |
| Observations | 3,136 | 3,091 | 3,091 | 3,091 | 3,086 | 2,990 |

*Notes*: Incidence rate ratios (IRR) are reported. t-statistics based on robust standard errors clustered at the state level are shown in parentheses. Regressors are entered in standardized form and each regression also included a constant. Specification (5) excludes New York City. Specification (6) excludes zero-death counties. *** p<0.01, ** p<0.05, * p<0.1.

**Table 4:** Covid-19 deaths and share of racial/ethnic group in county – As of March 15, 2021

|  | 1 | 2 | 3 | 4 | 5 | 6 |
| --- | --- | --- | --- | --- | --- | --- |
| Black | 1.142*** | 1.106*** | 1.110*** | 1.061*** | 1.060*** | 1.064*** |
|  | (5.849) | (4.942) | (3.057) | (3.862) | (3.859) | (4.072) |
| AIAN | 1.105*** | 1.162*** | 1.182*** | 1.102*** | 1.099*** | 1.103*** |
|  | (3.537) | (5.788) | (5.888) | (3.703) | (3.629) | (3.743) |
| Asian | 0.931*** | 0.963 | 1.006 | 1.035** | 1.026* | 1.035** |
|  | (-2.699) | (-1.605) | (0.284) | (2.574) | (1.869) | (2.561) |
| NHPI | 1.094** | 0.863* | 0.838** | 1.021 | 1.025 | 1.012 |
|  | (2.285) | (-1.897) | (-2.405) | (0.582) | (0.698) | (0.337) |
| Mixed | 0.816*** | 0.814*** | 0.830*** | 0.879*** | 0.881*** | 0.878*** |
|  | (-4.027) | (-4.899) | (-5.197) | (-5.088) | (-4.839) | (-4.837) |
| Hispanic | 1.065** | 1.078*** | 1.065** | 1.026 | 1.023 | 1.028 |
|  | (1.986) | (2.784) | (2.151) | (1.196) | (1.112) | (1.277) |
| Poverty |  |  | 0.990 | 1.002 | 1.000 | 1.001 |
|  |  |  | (-0.320) | (0.0812) | (-0.00648) | (0.0320) |
| Unemp. Rate |  |  | 0.920*** | 0.960** | 0.961** | 0.957*** |
|  |  |  | (-2.976) | (-2.375) | (-2.352) | (-2.663) |
| Per Capita Inc. |  |  | 0.912** | 0.874*** | 0.871*** | 0.874*** |
|  |  |  | (-2.526) | (-5.949) | (-6.155) | (-6.179) |
| No HS Diploma |  |  | 1.075* | 1.107*** | 1.102*** | 1.104*** |
|  |  |  | (1.653) | (4.715) | (4.694) | (4.555) |
| Age ≥ 65 |  | 1.205*** | 1.205*** | 1.189*** | 1.186*** | 1.197*** |
|  |  | (6.259) | (6.711) | (8.548) | (8.716) | (8.830) |
| Age ≤ 17 |  | 1.114*** | 1.108*** | 1.095*** | 1.098*** | 1.095*** |
|  |  | (3.403) | (3.489) | (6.264) | (6.250) | (6.662) |
| Uninsured Rate |  | 1.046 | 0.989 | 0.942*** | 0.942*** | 0.945*** |
|  |  | (1.413) | (-0.261) | (-2.915) | (-2.914) | (-2.842) |
| Pollution |  | 1.060*** | 1.056*** | 1.048** | 1.045** | 1.045* |
|  |  | (3.214) | (3.061) | (2.064) | (1.961) | (1.956) |
| alpha | 0.243*** | 0.203*** | 0.193*** | 0.122*** | 0.121*** | 0.117*** |
|  | (-13.43) | (-14.84) | (-14.01) | (-18.50) | (-18.45) | (-21.48) |
| State effects | No | No | No | Yes | Yes | Yes |
| Observations | 3,136 | 3,091 | 3,091 | 3,091 | 3,086 | 3,039 |

*Notes*: Incidence rate ratios (IRR) are reported. t-statistics based on robust standard errors clustered at the state level are shown in parentheses. Regressors are entered in standardized form and each regression also included a constant. Specification (5) excludes New York City. Specification (6) excludes zero-death counties. *** p<0.01, ** p<0.05, * p<0.1.

**Table 5:** Covid-19 deaths and largest racial/ethnic group in county – As of May 15, 2020

|  | | 1 | | 2 | | 3 | | 4 | | 5 | | 6 | |
| --- | --- | --- | --- | --- | --- | --- | --- | --- | --- | --- | --- | --- | --- |
| Black | | 3.547*** | | 2.899*** | | 2.690*** | | 2.307*** | | 2.325*** | | 1.861*** | |
|  | | (6.165) | | (4.937) | | (4.962) | | (5.745) | | (5.837) | | (4.141) | |
| AIAN | | 1.461 | | 6.017*** | | 5.361*** | | 6.227*** | | 6.036*** | | 5.139*** | |
|  | | (0.748) | | (3.283) | | (3.242) | | (3.169) | | (3.157) | | (3.730) | |
| ANHPI | | 0.340* | | 0.371*** | | 0.123*** | | 1.153 | | 1.102 | | 1.377** | |
|  | | (-1.881) | | (-4.770) | | (-6.039) | | (1.019) | | (0.625) | | (2.505) | |
| Hispanic | | 1.329 | | 1.324 | | 0.833 | | 0.934 | | 0.845 | | 0.958 | |
|  | | (0.589) | | (0.770) | | (-0.548) | | (-0.332) | | (-0.844) | | (-0.227) | |
| Poverty | |  | |  | | 1.357** | | 1.218** | | 1.217** | | 1.245*** | |
|  | |  | |  | | (2.398) | | (2.117) | | (2.093) | | (2.880) | |
| Unemp. Rate | |  | |  | | 1.040 | | 0.924 | | 0.924 | | 0.964 | |
|  | |  | |  | | (0.597) | | (-1.288) | | (-1.281) | | (-0.651) | |
| Per Capita Inc. | |  | |  | | 1.896*** | | 1.463*** | | 1.469*** | | 1.364*** | |
|  | |  | |  | | (4.993) | | (4.520) | | (4.428) | | (4.391) | |
| No HS Diploma | |  | |  | | 1.484*** | | 1.442*** | | 1.440*** | | 1.552*** | |
|  | |  | |  | | (3.410) | | (5.167) | | (5.147) | | (5.593) | |
| Age ≥ 65 | |  | | 0.780*** | | 0.838*** | | 0.953 | | 0.952 | | 1.155*** | |
|  | |  | | (-2.967) | | (-2.763) | | (-0.724) | | (-0.753) | | (2.738) | |
| Age ≤ 17 | |  | | 0.859* | | 0.889 | | 0.961 | | 0.966 | | 0.985 | |
|  | |  | | (-1.702) | | (-1.509) | | (-0.624) | | (-0.531) | | (-0.298) | |
| Uninsured Rate | |  | | 0.901 | | 0.896 | | 0.932 | | 0.930 | | 0.929 | |
|  | |  | | (-0.952) | | (-0.813) | | (-0.808) | | (-0.834) | | (-0.854) | |
| Pollution | |  | | 1.206*** | | 1.155*** | | 1.229*** | | 1.216*** | | 1.099** | |
|  | |  | | (4.586) | | (3.804) | | (4.185) | | (4.149) | | (2.021) | |
| alpha | | 2.475*** | | 2.161*** | | 1.967*** | | 1.355*** | | 1.354*** | | 0.719*** | |
|  | | (9.916) | | (10.31) | | (9.958) | | (4.156) | | (4.139) | | (-6.267) | |
| State effects | No | | No | | No | | Yes | | Yes | | Yes | |  |
| Observations | | 2,900 | | 2,866 | | 2,866 | | 2,866 | | 2,861 | | 1,613 | |

*Notes*: Incidence rate ratios (IRR) are reported. t-statistics based on robust standard errors clustered at the state level are shown in parentheses. ANHPI equals 1 if a county’s largest racial/ethnic group is Asian or NHPI, zero otherwise. Regressors (except racial/ethnic indicators) are entered in standardized form and each regression also included a constant. Specification (5) excludes New York City. Specification (6) excludes zero-death counties. *** p<0.01, ** p<0.05, * p<0.1.

**Table 6:** Covid-19 deaths and largest racial/ethnic group in county – As of August 15, 2020

|  | | 1 | | 2 | | 3 | | 4 | | 5 | | 6 | |
| --- | --- | --- | --- | --- | --- | --- | --- | --- | --- | --- | --- | --- | --- |
| Black | | 3.521*** | | 2.258*** | | 1.888*** | | 1.561*** | | 1.565*** | | 1.522*** | |
|  | | (13.28) | | (9.643) | | (3.980) | | (4.975) | | (4.992) | | (5.240) | |
| AIAN | | 2.070* | | 4.446*** | | 3.983*** | | 5.578*** | | 5.521*** | | 6.669*** | |
|  | | (1.869) | | (3.277) | | (3.566) | | (3.764) | | (3.764) | | (4.467) | |
| ANHPI | | 0.240*** | | 0.415*** | | 0.190*** | | 0.714*** | | 0.708*** | | 0.814** | |
|  | | (-2.868) | | (-5.894) | | (-6.567) | | (-3.280) | | (-3.154) | | (-2.496) | |
| Hispanic | | 2.063*** | | 1.839*** | | 1.157 | | 1.147 | | 1.113 | | 1.248** | |
|  | | (4.521) | | (3.019) | | (0.756) | | (1.084) | | (0.790) | | (2.240) | |
| Poverty | |  | |  | | 1.236** | | 1.169** | | 1.170** | | 1.180** | |
|  | |  | |  | | (2.533) | | (2.294) | | (2.289) | | (2.520) | |
| Unemp. Rate | |  | |  | | 1.076 | | 0.964 | | 0.965 | | 0.972 | |
|  | |  | |  | | (1.223) | | (-0.817) | | (-0.797) | | (-0.649) | |
| Per Capita Inc. | |  | |  | | 1.544*** | | 1.250*** | | 1.252*** | | 1.207*** | |
|  | |  | |  | | (5.068) | | (4.001) | | (3.912) | | (3.554) | |
| No HS Diploma | |  | |  | | 1.489*** | | 1.399*** | | 1.398*** | | 1.391*** | |
|  | |  | |  | | (4.859) | | (6.163) | | (6.088) | | (6.657) | |
| Age ≥ 65 | |  | | 0.926 | | 0.958 | | 1.016 | | 1.015 | | 1.115*** | |
|  | |  | | (-1.383) | | (-1.018) | | (0.401) | | (0.383) | | (3.520) | |
| Age ≤ 17 | |  | | 1.000 | | 1.017 | | 1.077** | | 1.079** | | 1.068** | |
|  | |  | | (-0.00125) | | (0.336) | | (2.138) | | (2.192) | | (2.326) | |
| Uninsured Rate | |  | | 1.159** | | 1.050 | | 0.959 | | 0.957 | | 0.916* | |
|  | |  | | (2.267) | | (0.541) | | (-0.749) | | (-0.788) | | (-1.830) | |
| Pollution | |  | | 1.206*** | | 1.158*** | | 1.196*** | | 1.190*** | | 1.141*** | |
|  | |  | | (7.064) | | (6.471) | | (6.661) | | (6.902) | | (4.805) | |
| alpha | | 1.247** | | 1.042 | | 0.947 | | 0.641*** | | 0.641*** | | 0.473*** | |
|  | | (2.158) | | (0.418) | | (-0.570) | | (-4.668) | | (-4.633) | | (-9.957) | |
| State effects | No | | No | | No | | Yes | | Yes | | Yes | |  |
| Observations | | 3,116 | | 3,116 | | 3,073 | | 3,073 | | 3,068 | | 2,355 | |

*Notes*: Incidence rate ratios (IRR) are reported. t-statistics based on robust standard errors clustered at the state level are shown in parentheses. ANHPI equals 1 if a county’s largest racial/ethnic group is Asian or NHPI, zero otherwise. Regressors (except racial/ethnic indicators) are entered in standardized form and each regression also included a constant. Specification (5) excludes New York City. Specification (6) excludes zero-death counties. *** p<0.01, ** p<0.05, * p<0.1.

**Table 7:** Covid-19 deaths and largest racial/ethnic group in county – As of December 15, 2020

|  | | 1 | | 2 | | 3 | | 4 | | 5 | | 6 | |
| --- | --- | --- | --- | --- | --- | --- | --- | --- | --- | --- | --- | --- | --- |
| Black | | 1.815*** | | 1.675*** | | 1.554*** | | 1.231*** | | 1.240*** | | 1.250*** | |
|  | | (9.589) | | (8.705) | | (3.817) | | (4.045) | | (4.233) | | (4.381) | |
| AIAN | | 1.968*** | | 1.982*** | | 2.419*** | | 2.061*** | | 2.020*** | | 2.050*** | |
|  | | (3.034) | | (3.075) | | (3.681) | | (2.636) | | (2.588) | | (2.676) | |
| ANHPI | | 0.209*** | | 0.445*** | | 0.448*** | | 1.190*** | | 1.201*** | | 1.175*** | |
|  | | (-4.834) | | (-10.33) | | (-4.563) | | (3.037) | | (3.254) | | (2.868) | |
| Hispanic | | 1.535** | | 1.378** | | 1.166 | | 1.198*** | | 1.165** | | 1.207*** | |
|  | | (2.505) | | (2.015) | | (1.021) | | (2.637) | | (1.983) | | (2.663) | |
| Poverty | |  | |  | | 1.091* | | 1.067* | | 1.059 | | 1.067 | |
|  | |  | |  | | (1.782) | | (1.675) | | (1.500) | | (1.642) | |
| Unemp. Rate | |  | |  | | 0.900*** | | 0.946*** | | 0.947** | | 0.942*** | |
|  | |  | |  | | (-2.648) | | (-2.592) | | (-2.554) | | (-2.829) | |
| Per Capita Inc. | |  | |  | | 0.994 | | 0.938* | | 0.927** | | 0.937* | |
|  | |  | |  | | (-0.104) | | (-1.724) | | (-2.384) | | (-1.714) | |
| No HS Diploma | |  | |  | | 1.156** | | 1.199*** | | 1.188*** | | 1.196*** | |
|  | |  | |  | | (2.466) | | (5.847) | | (6.189) | | (5.901) | |
| Age ≥ 65 | |  | | 1.214*** | | 1.200*** | | 1.175*** | | 1.175*** | | 1.190*** | |
|  | |  | | (6.482) | | (6.708) | | (6.663) | | (6.681) | | (6.955) | |
| Age ≤ 17 | |  | | 1.184*** | | 1.177*** | | 1.125*** | | 1.134*** | | 1.123*** | |
|  | |  | | (4.021) | | (4.108) | | (6.050) | | (6.064) | | (5.785) | |
| Uninsured Rate | |  | | 1.075 | | 0.974 | | 0.931*** | | 0.929*** | | 0.930*** | |
|  | |  | | (1.501) | | (-0.400) | | (-2.848) | | (-2.962) | | (-3.112) | |
| Pollution | |  | | 1.045* | | 1.035 | | 1.058** | | 1.050** | | 1.051* | |
|  | |  | | (1.919) | | (1.493) | | (2.015) | | (1.977) | | (1.795) | |
| Alpha | | 0.451*** | | 0.406*** | | 0.388*** | | 0.236*** | | 0.234*** | | 0.223*** | |
|  | | (-8.116) | | (-9.102) | | (-8.297) | | (-13.79) | | (-14.22) | | (-15.65) | |
| State effects | No | | No | | No | | Yes | | Yes | | Yes | |  |
| Observations | | 3,136 | | 3,091 | | 3,091 | | 3,091 | | 3,086 | | 2,990 | |

*Notes*: Incidence rate ratios (IRR) are reported. t-statistics based on robust standard errors clustered at the state level are shown in parentheses. ANHPI equals 1 if a county’s largest racial/ethnic group is Asian or NHPI, zero otherwise. Regressors (except racial/ethnic indicators) are entered in standardized form and each regression also included a constant. Specification (5) excludes New York City. Specification (6) excludes zero-death counties. *** p<0.01, ** p<0.05, * p<0.1.

**Table 8:** Covid-19 deaths and largest racial/ethnic group in county – As of March 15, 2021

|  | | 1 | | 2 | | 3 | | 4 | | 5 | | 6 | |
| --- | --- | --- | --- | --- | --- | --- | --- | --- | --- | --- | --- | --- | --- |
| Black | | 1.538*** | | 1.357*** | | 1.272*** | | 1.101** | | 1.106*** | | 1.108*** | |
|  | | (7.784) | | (6.246) | | (2.892) | | (2.505) | | (2.647) | | (2.672) | |
| AIAN | | 1.598** | | 1.912*** | | 2.234*** | | 1.750*** | | 1.726*** | | 1.752*** | |
|  | | (2.139) | | (3.019) | | (3.984) | | (2.838) | | (2.777) | | (2.860) | |
| ANHPI | | 0.289*** | | 0.681*** | | 0.769** | | 1.422*** | | 1.433*** | | 1.419*** | |
|  | | (-3.044) | | (-7.262) | | (-2.110) | | (11.22) | | (12.67) | | (11.66) | |
| Hispanic | | 1.430*** | | 1.368*** | | 1.170* | | 1.084 | | 1.068 | | 1.088 | |
|  | | (3.052) | | (3.620) | | (1.901) | | (1.603) | | (1.250) | | (1.642) | |
| Poverty | |  | |  | | 1.018 | | 1.019 | | 1.014 | | 1.019 | |
|  | |  | |  | | (0.477) | | (0.688) | | (0.514) | | (0.663) | |
| Unemp. Rate | |  | |  | | 0.929** | | 0.962** | | 0.962** | | 0.959** | |
|  | |  | |  | | (-2.414) | | (-2.325) | | (-2.314) | | (-2.564) | |
| Per Capita Inc. | |  | |  | | 0.928* | | 0.889*** | | 0.881*** | | 0.889*** | |
|  | |  | |  | | (-1.704) | | (-4.670) | | (-6.023) | | (-4.727) | |
| No HS Diploma | |  | |  | | 1.132*** | | 1.141*** | | 1.133*** | | 1.138*** | |
|  | |  | |  | | (2.850) | | (6.314) | | (6.606) | | (6.220) | |
| Age ≥ 65 | |  | | 1.234*** | | 1.213*** | | 1.184*** | | 1.184*** | | 1.192*** | |
|  | |  | | (7.842) | | (8.276) | | (9.479) | | (9.516) | | (9.774) | |
| Age ≤ 17 | |  | | 1.132*** | | 1.124*** | | 1.098*** | | 1.103*** | | 1.098*** | |
|  | |  | | (3.528) | | (3.565) | | (6.199) | | (6.206) | | (6.488) | |
| Uninsured Rate | |  | | 1.102** | | 1.002 | | 0.943*** | | 0.942*** | | 0.946*** | |
|  | |  | | (2.320) | | (0.0335) | | (-2.653) | | (-2.704) | | (-2.578) | |
| Pollution | |  | | 1.066*** | | 1.056*** | | 1.049** | | 1.044* | | 1.047* | |
|  | |  | | (3.606) | | (3.046) | | (2.041) | | (1.910) | | (1.952) | |
| alpha | | 0.294*** | | 0.236*** | | 0.218*** | | 0.127*** | | 0.126*** | | 0.122*** | |
|  | | (-11.74) | | (-14.30) | | (-12.77) | | (-17.95) | | (-18.00) | | (-20.73) | |
| State effects | No | | No | | No | | Yes | | Yes | | Yes | |  |
| Observations | | 3,136 | | 3,091 | | 3,091 | | 3,091 | | 3,086 | | 3,039 | |

*Notes*: Incidence rate ratios (IRR) are reported. t-statistics based on robust standard errors clustered at the state level are shown in parentheses. ANHPI equals 1 if a county’s largest racial/ethnic group is Asian or NHPI, zero otherwise. Regressors (except racial/ethnic indicators) are entered in standardized form and each regression also included a constant. Specification (5) excludes New York City. Specification (6) excludes zero-death counties. *** p<0.01, ** p<0.05, * p<0.1.
